# The nature and persistence of the effects of posthypnotic suggestions on food preferences: The final report of an online study

**DOI:** 10.3389/fpsyg.2023.1123907

**Published:** 2023-03-22

**Authors:** Anoushiravan Zahedi, Renin Öznur Akalin, Johanna E. Lawrence, Annika Baumann, Werner Sommer

**Affiliations:** ^1^Department of Psychology, Humboldt-Universität zu Berlin, Berlin, Germany; ^2^Department of Decision Neuroscience and Nutrition, German Institute of Human Nutrition (DIfE), Nuthetal, Germany; ^3^Neuroscience Research Center, Charité-Universitätsmedizin Berlin, Corporate Member of Freie Universität Berlin, Humboldt-Universität zu Berlin, and Berlin Institute of Health, Neuroscience Research Center, Berlin, Germany; ^4^Department of Psychology, University of Muenster (Westfaelische Wilhelms-Universitaet Muenster), Münster, Germany; ^5^Berlin School of Mind and Brain, Humboldt-Universität zu Berlin, Berlin, Germany; ^6^Weizenbaum Institute for the Networked Society, Berlin, Germany; ^7^University of Potsdam, Chair of Business Informatics, esp. Social Media and Society, Potsdam, Germany

**Keywords:** hypnosis, online supermarket, posthypnotic suggestions (PHSs), food choice, eating behavior, food preferences, Bayesian analysis, Bayesian generalized linear mixed model

## Abstract

The persistence of food preferences, which are crucial for diet-related decisions, is a significant obstacle to changing unhealthy eating behavior. To overcome this obstacle, the current study investigates whether posthypnotic suggestions (PHSs) can enhance food-related decisions by measuring food choices and subjective ratings. After assessing hypnotic susceptibility in Session 1, at the beginning of Session 2, a PHS was delivered aiming to increase the desirability of healthy food items (e.g., vegetables and fruit). After the termination of hypnosis, a set of two tasks was administrated twice, once when the PHS was activated and once deactivated in counterbalanced order. The task set consisted of rating 170 pictures of food items, followed by an online supermarket where participants were instructed to select enough food from the same item pool for a fictitious week of quarantine. After 1 week, Session 3 mimicked Session 2 without renewed hypnosis induction to assess the persistence of the PHS effects. The Bayesian hierarchical modeling results indicate that the PHS increased preferences and choices of healthy food items without altering the influence of preferences in choices. In contrast, for unhealthy food items, not only both preferences and choices were decreased due to the PHS, but also their relationship was modified. That is, although choices became negatively biased against unhealthy items, preferences played a more dominant role in unhealthy choices when the PHS was activated. Importantly, all effects persisted over 1 week, qualitatively and quantitatively. Our results indicate that although the PHS affected healthy choices through resolve, i.e., preferred more and chosen more, unhealthy items were probably chosen less impulsively through effortful suppression. Together, besides the translational importance of the current results for helping the obesity epidemic in modern societies, our results contribute theoretically to the understanding of hypnosis and food choices.

## 1. Introduction

The increasingly obesogenic prevalent diets (Swinburn et al., 1999; Jaacks et al., 2019; Clark et al., 2022) in modern society (e.g., high in sugar or salt, high-fat red meat, ultra-processed food, “junk food”) are posing threats to human health, biodiversity, and the climate. Therefore, there is an urgent need to shift toward more healthy diets (e.g., Willett et al., 2019). The rampant obesity epidemic demonstrates that traditional efforts toward diet change are insufficient (Pereira et al., 2005; Navarro-Allende et al., 2008; Kakoschke et al., 2017; Jones et al., 2018). Therefore, it is crucial to seek new ways to strengthen healthy food choices. Notably, food choices are subject to several interacting factors: food preferences, impulsive reactions, and cognitive control (Guerrieri et al., 2008; Nederkoorn et al., 2009, 2010; Bongers et al., 2015). Often, good intentions to eat healthy food disintegrate under the force of competing preferences or impulsive behavior. The traditional approach to diet regulations focuses mainly on unhealthy food restrictions through strengthening cognitive control, which showed limited success at best (for review, see Stephens et al., 2014; Yang et al., 2019). In the present study, we explore an alternative strategy and investigate the utility of posthypnotic suggestions (PHSs) in biasing food preferences in favor of a healthier diet.

Improving diet habits, which are already formed during sensitive periods early in life (Wilson, 2015; Maier-Noth, 2019), requires increasing the preference for and desirability of healthy food on an affective level (Zahedi et al., 2020a). The acquisition and modulation of food preferences and eating habits involve congenital factors, exposure (Bornstein, 1989), and a multitude of cognitive (Yang et al., 2019), affective (Zahedi et al., 2020a), social, and cultural influences (Enriquez and Archila-Godinez, 2021) that no single intervention can shoulder. However, PHSs can integrate cognitive and psychosocial factors and successfully change implicit food preferences toward more healthy options (Ludwig et al., 2014; Zahedi et al., 2020a). Nevertheless, previous efforts were (1) mainly focused on food preferences and not on actual food choices, (2) did not investigate the persistence of the effects, and (3) only recruited participants who were at least moderately responsive to hypnotic suggestions. These issues are addressed in the present study.

To better estimate the effects of PHSs in real-life-like situations, we utilized (I) an online supermarket mockup that included a large number of food items, and (II) measured subjective values for the same items. By measuring both subjective values and food choices, we were able to calculate choice-preference relationships. Choice-preference relationships in binary choices were analyzed using logistic regression modeling (McKerchar et al., 2009; Peng et al., 2010; Scherbaum et al., 2012). Choice-preference functions inform about choice biases (i.e., intercepts in the model) and dependencies of choices on preferences (i.e., slopes in the model). These results can be used to shed light on the underlying cognitive mechanisms of choice behavior. Additionally, (III) in order to address whether the effects persist over time, we re-tested the effects of the PHS after 1 week. Finally, (IV) to assess the generalizability of the previous results (Zahedi et al., 2020a), we recruited participants regardless of their responsiveness to hypnotic suggestions.

### 1.1. Hypothesis

Together, food choices, preferences, and choice-preference functions can be used to elucidate the mechanisms underlying the PHS effects. If choices and preferences for healthy food items are increased in the PHS-activated compared to the PHS-deactivated condition, but if the choice-preference function is unaffected, one can conclude that the PHS modulates choices by affecting explicit preferences. In contrast, if choices of healthy food items are increased but preferences are not, then a decrease in the choice-preference function's slopes or a positive choice bias may indicate that the PHS affects implicit food preferences that are not explicitly accessible. Finally, the increase in preferences without any modulation of choices but accompanied by increased slopes of the choice-preference function or induction of negative bias for healthy items indicates that the PHS can only affect explicit preferences that are insufficient for affecting choices.

Concerning unhealthy food items, if preferences and choices are decreased, a stable choice-preference function indicates that the PHS modulates choices by affecting explicit food preferences. In contrast, if choices of unhealthy food items are reduced but not preferences, an increase in slopes of the choice-preference function and/or a negative choice bias should be expected. This can be interpreted as related to an increased contribution of top-down cognitive control in food choices. Notably, for unhealthy food items, we expect any decrease in preferences to be accompanied by a decrease in choices.

Furthermore, we expected the PHS effects on food choice and food preferences to be stable across sessions. Finally, participants' hypnotizability should be correlated with the observed behavioral effects.

## 2. Materials and methods

### 2.1. Participants and inclusion criteria

Of the respondents to our advertisements, 55 (43 female, *mean Age* = 26.9 [19 − 39] *years*±6.03) were recruited in the study, of which 50 (38 female, *mean Age* = 26.6 [19 − 39] *years*±5.70) completed all three sessions. The minimum sample size of 40 participants had been based on a priori power analysis with $\alpha <0.05,\;1-\beta >0.95,\;\eta_{p}^{2}>0.08$. The critical values were determined based on the suggestion of Cohen (2016), and the effect size was based on previous results (e.g., Zahedi et al., 2020a). Notably, Zahedi et al. (2020a) found a medium effect size of $\eta_{p}^{2}\;=\;0.22$. However, since only medium- and high-hypnotizable participants were included in that sample, we adjusted the expected effect size for the current study from medium, i.e., $\eta_{p}^{2}\;=\;0.22$, to small, i.e., $,\;\eta_{p}^{2}\;=\;0.08$. This adjustment ascertained that in the current study, where participants were included regardless of their hypnotizability scores, we have the statistical power to detect possible effects. Notably, the a priori power analysis, in tandem with the Bayesian statistics used, gives us the necessary tools to interpret possible null results appropriately, as well.

The exclusion criteria were being either underweight (BMI <18) or obese (BMI > 30), or having a history of psychological or neurological problems. The criteria for healthy body weight were selected based on the recommendation of WHO (2022). However, all volunteers met the inclusion criteria, and therefore, no one had to be excluded (*mean BMI was* 22.0 [18.0 − 27.1]±2.40). The study was approved by the ethics committee of the Department of Psychology of the Humboldt-Universität zu Berlin (approval number 2021-36). Prior to the experiment, informed consent was obtained according to the declaration of Helsinki, and participation was compensated with 10 Euro/hour (*N* = 25) or course credits (*N* = 30). The study was conducted fully online.

### 2.2. Materials and tasks

The hypnotizability of participants was measured by the German version (Bongartz, 1985) of the Harvard group scale of hypnotic susceptibility—form A (HGSHS: A; Shor and Orne, 1962). In HGSHS: A, 12 different suggestions are delivered to participants, and their responsiveness is determined based on the number of items to which they could respond (based on self-reports). According to the scoring procedure suggested by Kihlstrom and Register (1984), scores between 0 and 12 can be achieved.

Other questionnaires to be completed were the Edinburgh Handedness Questionnaire (EHQ; Oldfield, 1971), the German Nutrition Knowledge Questionnaire (NKQ; De Souza et al., 2015), and the Self-Regulation of Eating Behavior Questionnaire (SREBQ; Kliemann et al., 2016). EHQ consists of 20 questions, evaluating which hand is usually used for administrating specific tasks, such as writing or throwing. The NKQ consists of 22 questions about the knowledge of healthy food choices and the sources of nutrients in food. The SREBQ consists of four questions aiming to evaluate an individual's capacity for regulating their eating behavior.

**Figure 1:**
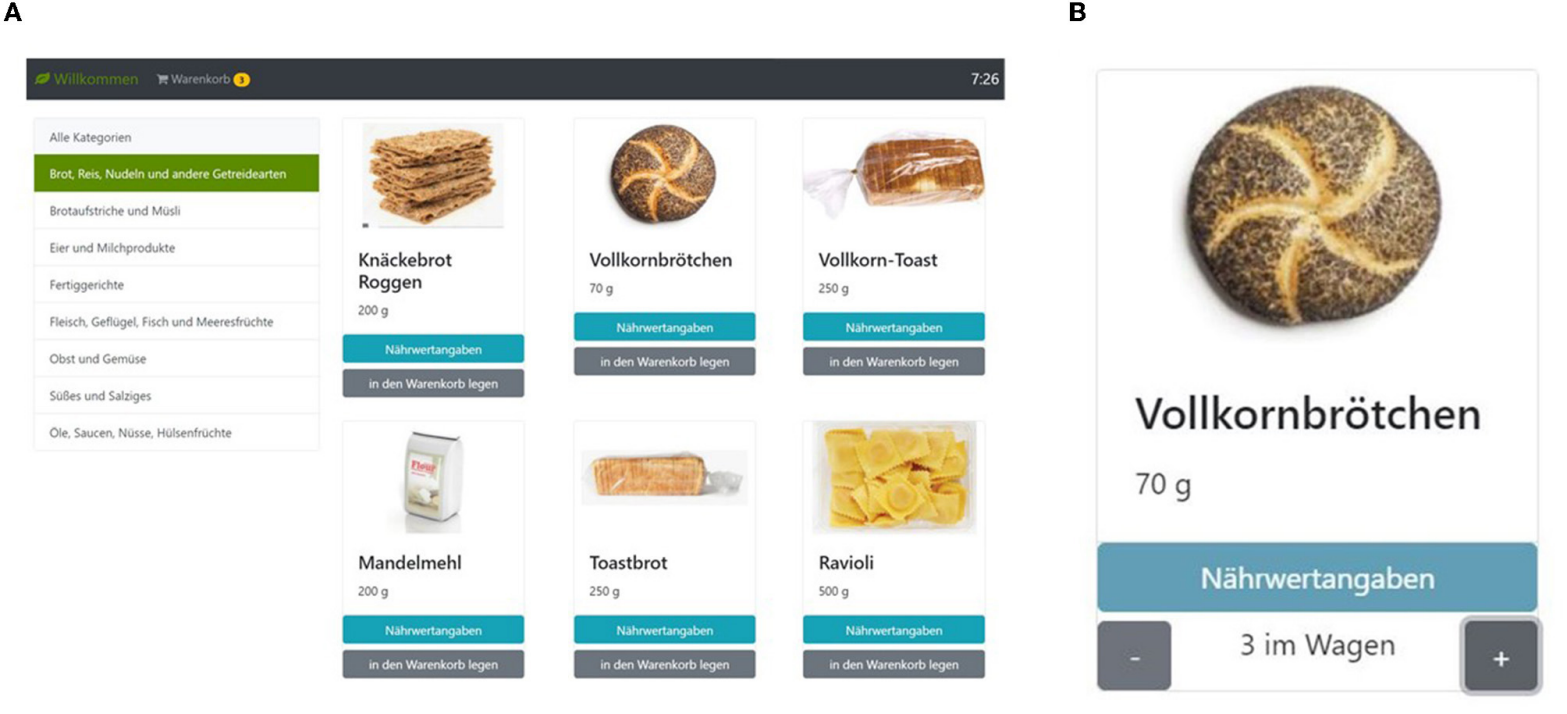
The screenshot of the supermarket task. **(A)** Participants could choose different food categories shown on the left side to see the items belonging to the respective category (in the example, the category ‘bread, rice, pasta, and other grain products' has been selected). On the top right corner, the remaining shopping time is shown; on the top left, they can see their shopping basket (“Warenkorb”), which can be selected to modify its contents and place the order. **(B)** For each object, participants can choose to inspect the nutritional values (“Nährwertangaben”) and select how many exemplars of the item they wish to put in their basket. The shopping task is publicly available for non-commercial use; please see Supplementary material for further information.

The online supermarket (Figure 1) was based on eight food categories, including 170 products in total. The organization and items were inspired by existing online shops and aimed to simulate real-life online food shopping behavior. For instance, a diverse array of options was presented for each product (i.e., full-fat and low-fat milk) to enable participants to choose their preferred items. The eight categories of food items in the supermarket are as follows:

1. Bread, rice, pasta, and other grain products (e.g., toast bread, pretzel, croissant),
2. Bread spreads and breakfast cereals (e.g., honey, jams, chocolate creams),
3. Eggs and dairy (e.g., milk, cheese, yogurt),
4. Convenience foods (e.g., filled pasta, pizza, potato salad),
5. Meat, poultry, fish, seafood (e.g., salami, minced meat, smoked salmon),
6. Fruits and vegetables (e.g., tomato, onion, pepper),
7. Sweets and salty snacks (e.g., chocolate, candy, ice cream, potato chips),
8. Oils, sauces, nuts, legumes (e.g., olive oil, cashew nuts, ketchup)

After choosing a food category, between 16 and 26 images per category were shown with the name underneath. Further, the nutrition facts for each item could be inspected by clicking a corresponding button on the screen (“Nährwertangaben”). Each item could be placed in the shopping basket by pressing a corresponding button on the screen (“in den Warenkorb legen”). The package sizes per item were relatively small, equal to approximately one serving; for example, participants could choose to buy a single egg or a single potato. However, there was no limit to the number of a given item that could be placed in the shopping basket. Also, participants could directly select a specific number (i.e., 1 ≤ *n* ≤ 20) of each item. The shopping basket could be inspected to correct the number of items in the basket before placing the final order.

The online supermarket was introduced with the instructions that participants should imagine that they have to quarantine for 1 week. All food they wanted to consume during this period had to be ordered from the online supermarket. They had no budget limit and could choose as many products as desired. The only restriction was the time limit of 15 min for the supermarket task. The shopping task is publicly available for non-commercial use; please see Supplementary material for further information.

In the food preference rating task, participants were shown all the food items offered in the supermarket task in randomized order. Participants were to rate each item for how much they liked it in principle, independently of whether they wanted it at the moment. Ratings were performed on a Likert scale from 1 (Don't like it at all) to 7 (Like it very much). There was a response window of 20 s for each item, after which the trial was considered a miss. The food preference rating task required about 10 min.

### 2.3. Procedure

The experiment was conducted online *via* the Zoom platform and involved three sessions. All questionnaires were implemented through the SoSci Survey platform (Version 3.0. 01, www.soscisurvey.de), and the individualized links were sent to participants in real time during each session. In Session 1, written informed consent was obtained, and demographic information (i.e., age, sex, height, weight, educational background), NKQ, and SREBQ were collected. Afterward, the German version of HGSHS: A was administered to determine the hypnotic susceptibility of participants. We did not exclude any participants based on the screening results. Instead, susceptibility scores were used as a regressor in subsequent analyses. Session 1 took about 2 h and was conducted as an online group session with up to five participants. About 1 week (*mean* = 8.51 ± 1.26 *days*) after Session 1, Session 2 was conducted, lasting about 2 h, followed by Session 3 after 3–10 days (*mean* = 6.24 ± 2.28 *days*), which took about 1 h. In Sessions 2 and 3, participants were tested individually. Session 2 started with hypnosis that included a PHS aiming to induce a strong desire for healthy food. The hypnosis procedure and the employed PHS (for details, see Appendix A) were the same as in Zahedi et al. (2020a). Next, the food preference rating and the online supermarket were administrated twice, once with the PHS activated and once deactivated. The order of conditions (i.e., PHS activated and deactivated) was counterbalanced across participants. Session 3 was identical in its procedure to Session 2, except that no hypnosis was applied. The order of PHS activation and deactivation for each participant was the same as in Session 2.

### 2.4. Data analysis

Based on our previous results (Zahedi et al., 2020a), we expected that posthypnotic suggestions would increase subjective preferences for healthy food items and decrease the subjective preferences for unhealthy food without affecting the choice-preference function. That means participants choose what they want based on the same principles as before, and therefore, the choice-preference function is unaltered. Thus, if preferences for healthy food items are increased, participants will choose more healthy food while the choice-preference function remains the same. Alternatively, choices may change, although preferences have not. In this case, the choice-preference function will also be affected, indicating that other mechanisms, such as increased suppression of temptation, must be considered as the driver of the changes. Finally, if preferences or choices of neutral food items (i.e., control items) were modulated, it reveals the opportunistic strategy used in response to experimental manipulation.

Independently from food categories, online supermarket items were categorized regarding their healthiness. Following Clark et al. (2019), we categorized the following items as healthy: (1) vegetables, (2) fruits, (3) legumes, and (4) some fish and marine products. Unhealthy food items were: (1) red meat, (2) processed and ultra-processed food, and (3) sugary and salty snacks. The choice set also contained items neither belonging to the healthy nor unhealthy food category and represented neutral items, used as our control items in subsequent analyses. The complete list of food items and their healthiness category can be found in the Supplementary material.

By conducting Bayesian generalized linear modeling, we investigate whether the PHS condition and its interaction with health categories and time (i.e., Session 2 or Session 3) affected the targeted outcome. Two main outcomes were the focus of our analyses: subjective food preferences, as measured by the food rating task, and food choices, as measured by the online shopping task. Depending on the outcome in focus, the models are either denoted as a Preference Model or Choice Model. In each model, the PHS condition (PHS-activated vs. PHS-deactivated), Session (Session 2 vs. Session 3), Healthiness of food items (healthy, neutral, and unhealthy), and the interaction between these factors were included as fixed effects. The intercept for all models was the healthy category, PHS-deactivated, Session 2. Further, three random effects were assumed: (1) a random intercept for the participants, (2) a random intercept for food items, and (3) a random slope for participants' hypnotizability on the PHS and Healthiness interaction (Model 1; Equation 1).


(1) $$\begin{array}{l} Model\;1:\;Outcome\;\sim \;Session*PHS*Healthiness+\left(1|Subjects\right)\\ \;+\left(1|Food\;Items\right)+\left(0+PHS*Healthiness\;|Hypnotizability\right) \end{array}$$

Three additional models were compared to the full model (Model 1) to gauge whether adding each factor improved the model's predictive capability: a model with only random intercepts (Model 4; Equation 4), a model with random intercepts and the fixed effect of Healthiness (Model 3; Equation 3), and a model with random intercepts and slope and the fixed effects of PHS and Healthiness (Model 2; Equation 2).


(2) $$\begin{array}{l} Model\;2:\;Outcome\;\sim \;PHS*Healthiness+\left(1|Subjects\right)\\ +(1|Food\;Items)+(0+PHS*Healthiness|Hypnotizability) \end{array}$$

(3) $$\begin{array}{l} Model\;3:\;Outcome\;\sim \;Healthiness+(1|Subjects)\\ \;+(1|Food\;Items) \end{array}$$

(4) $$\begin{array}{l} Model\;4:\;Outcome\;\sim \;1+\left(1|Subjects\right) \end{array}$$

Further, when a significant behavioral result was observed, we tested the Bayesian equivalent of the robust correlation between the observed effects and the hypnotizability scores.

The results of the food rating and the online supermarket tasks were used to calculate logistic regression models (McKerchar et al., 2009; Scherbaum et al., 2012). For calculating these choice-preference functions, choices were entered into the model as binary input (i.e., yes = 1, no = 0) and subjective ratings as continuous predictors. The output of the model represents the probability of choosing an item, given the subjective rating for that item:


(5) $$p_{j,i,k}(Y)=\frac{1}{1+\exp^{(\beta_{0}\sum_{i}\beta_{i}x_{i})}},$$

where *x* designates subjective rating, *Y* choice, *j* participant number, *i* session, *k* food category, and β_0_ and β_*l*_ are model parameters. The choice-preference functions were analyzed with the same approach used for assessing subjective ratings and food choices. The only difference is that subjective food ratings will always be used as a regressor in the models (from the baseline model to the full model). Further, the outcome will be a binary choice variable for each item, condition, and participant rather than the number of chosen items, which was used in Choice Models (Equation 6).


(6) $$\begin{array}{l} Choice_{Binary}\sim \;Preferences*Session*PHS*Healthiness\\ \;+\left(1|Subjects\right)+(1|Food\;Items)\\ \;+(0+PHS*Healthiness|Hypnotizability) \end{array}$$

All statistical analyses were conducted using the R programming language (http://www.R-project.org/). For calculating Bayesian hierarchical generalized linear models, brms (Bürkner, 2017) and RSTan (https://mc-stan.org/) were employed. The robust Bayesian correlations were calculated using RStan (https://mc-stan.org/). As all the models were multilevel, uninformative priors were preferred (Bürkner, 2017). Hence, we used *N*(0, 2.5) as uninformative priors in the models for β coefficients, *student*−*t*(3, 0, 2.5) for standard deviations, and *gamma*(0.01, 0.01) for shape when necessary. Subjective food ratings, food choices, and choice-preference functions were modeled using cumulative, negative binomial, and logistic families, respectively. All models were calculated with ten chains, each having 5,000 iterations with 1,000 warmups. If any variable showed a *Rhat* (i.e., the potential scale reduction factor on split chains) above 1.05, the model was recalculated with increased iterations and reported accordingly. For the model comparison, we used the Pareto smoothed importance sampling (PSIS) estimation of leave-one-out cross-validation (loo) implemented in the loo package (Vehtari et al., 2016; Magnusson et al., 2020).

All hypotheses were tested using the hypothesis package from brms (Bürkner, 2017). Based on the suggestion of van Doorn et al. (2021), *Bayes factors*(*BF*)>3 were considered as significant evidence for the tested hypothesis. One-sided hypotheses (*BF*_+0_ and *BF*_0+_) were the comparison of the posterior probability of hypotheses against their alternative; two sided-tests (*BF*_10_ and *BF*_01_) were the comparison between hypotheses and their alternative computed *via* the Savage-Dickey density ratio method.

## 3. Results

### 3.1. Subjective food preferences

**Figure 2:**
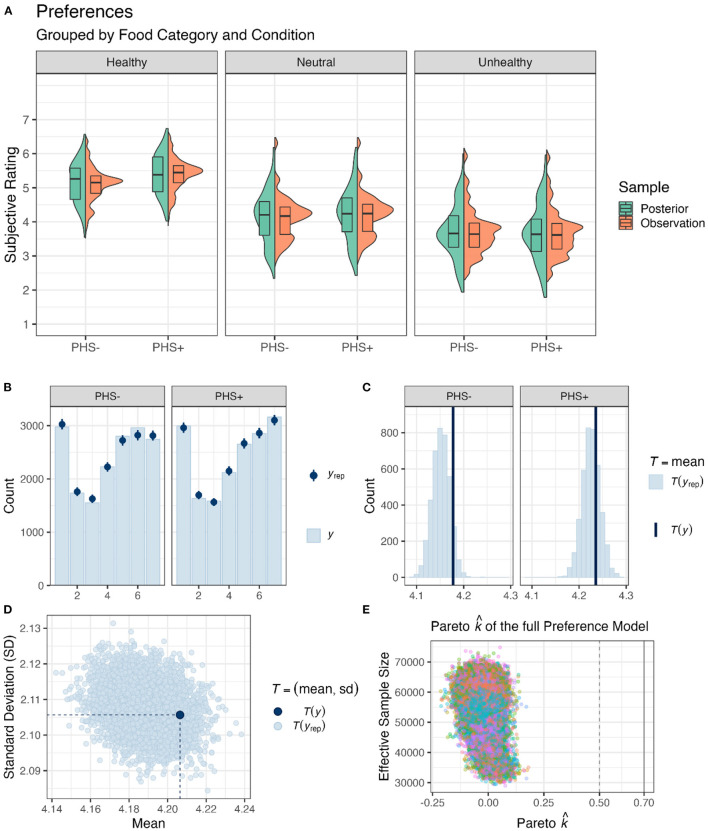
Food preferences. **(A)** Box and violin plots of the average preferences per participant for different food categories and PHS conditions pooled over Session. Green shapes depict observed data, and orange ones are from posterior distribution derived from the full Preference Model. **(B)** Means and standard error estimations, obtained from posterior distributions of preference ratings (*y*_*rep*_) per level of the scale in both PHS conditions, are depicted by dark blue points and lines; light blue bars depict means of observed data (*y*). **(C)** Frequency density bar plots showing the estimated (*y*_*rep*_) and observed mean preferences (*y*) in both PHS conditions. **(D)** A scatter plot showing the diffusion of subjective ratings. Light blue dots (*y*_*rep*_) are estimated based on posterior distributions, and the dark blue dot (*y*) is the observed value. **(E)** Pareto $\hat{k}$ values for the full Prefences Model are depicted against the effective sample sizes for importance sampling.

First, to investigate the effectiveness of our PHS, we analyzed participants' subjective preferences (Figure 2A). The full Preference Model (Equation 1) tested the effect of PHS, Session, and Healthiness on subjective food ratings. The full Preference Model (Equation 1) had no divergent transition, all *Rhat* = 1.00, and all variables had bulk- and tail-effective sample sizes >2,000 and >5,000. Posterior checks showed that the cumulative count model simulations reasonably captured the features of the observed data, including distributions (Figure 2B), means of different conditions (Figure 2C), and dispersion (Figure 2D).

**Figure 3:**
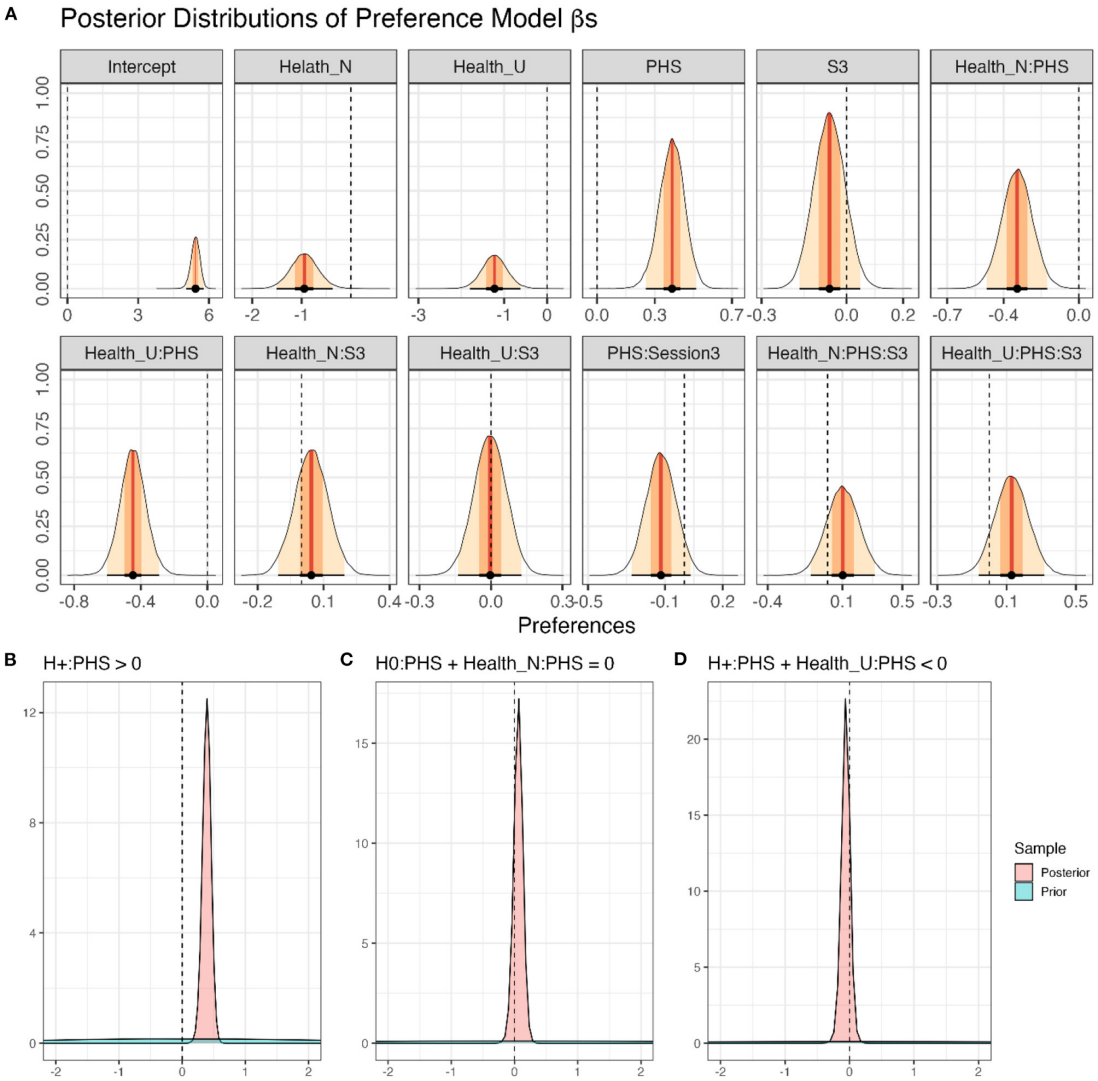
Modeling of food preferences. **(A)** Density plots of posterior distributions of all β-coefficients included in the full Preference Model (Model 1; Equation 1). The presented values are back-transformed from the cumulative logit scale. Red lines and orange and yellow shadows represent the mean point estimates and the 50 and 95% highest probability density (HPD) areas, respectively. Please note the different scalings of the X-axes. **(B–D)** The posterior distributions for specific a priori hypotheses. For more details regarding the hypotheses and results, see the text.

After confirming the validity of the Preference Model, the posteriors drawn from it were used to test our hypotheses (Figure 3). The results showed that activating PHS (PHS+) increased preferences for healthy food items (*H*_+_:*ConditionPHS*>0; *mean* = 0.39[0.28, 0.49], *sd* = 0.07, *p*.*p*.>0.99, **BF**_**+0**_**>9999**). Further, activating PHS did not affect the preferences for neutral items (*H*_0_:*ConditionPHS*+ *FoodItemHealthN*:*ConditionPHS* = 0; *mean* = 0.06[−0.8, 0.19], *sd* = 0.08, *p*.*p*. = 0.97, **BF**_**01**_** = 33.65**). Further, activating PHS probably decreased preferences for unhealthy items (*H*_+_:*ConditionPHS*+ *FoodItemHealthU*:*ConditionPHS* < 0; *mean* = −0.06[−0.16, 0.04], *sd* = 0.06, *p*.*p*. = 0.84, **BF**_**+0**_** = 5.19**). Notably, although the results indicate that preferences for healthy food items were increased by PHS+, the evidence supporting a concomitant decrease in preferences for unhealthy food items is not strong. Given that zero is within the 95% confidence interval of alterations in unhealthy food preferences due to PHS, one should interpret this result with caution. Hence, although our results indicate that unhealthy food items' preferences were more likely (*p*.*p*. = 0.83) to be decreased rather than increased (*p*.*p*. = 0.17) due to PHS, one cannot rule out the possibility of no alterations.

Factor Session affected subjective food preference ratings neither as a main effect (*H*_0_:*SessionS*3 = 0;*mean* = −0.06[−0.17, 0.05], *sd* = 0.05, *p*.*p*. = 0.96, **BF**_**01**_** = 25.12**), nor in interaction with PHS (*H*_0_:*SessionS*3:*ConditionPHS* = 0;*mean* = −0.12[−0.27, 0.03], *sd* = 0.08, *p*.*p*. = 0.91, **BF**_**01**_** = 10.06**), food category (*H*_0_:*SessionS*3:*FoodItemHealthN*+*SessionS*3:*FoodItemHealthU* = 0; *mean* = 0.04[−0.21, 0.29], *sd* = 0.13, *p*.*p*. = 0.96, **BF**_**01**_** = 25.61**), or in interaction with both PHS and food category (*H*_0_:*SessionS*3:*ConditionPHS*:*FoodItemHealthN*+*SessionS*3:*ConditionPHS*:*FoodItemHealthU* = 0; *mean* = 0.23[−0.13, 0.59], *sd* = 0.18, *p*.*p*. = 0.90, **BF**_**01**_** = 8.83**).

Finally, healthy food items were preferred more than neutral items (*H*_+_:*FoodItemHealthN* < 0; *mean* = −0.94[−1.41, −0.47], *sd* = 0.29, *p*.*p*.>0.99, **BF**_**+0**_** = 644.16**), and more than unhealthy items (*H*_+_:*FoodItemHealthU* < 0; *mean* = −1.22[−1.71, −0.73], *sd* = 0.30, *p*.*p*.>0.99, **BF**_**+0**_** = 3332.33**). Also, neutral food items were probably preferred more than unhealthy items (*H*_+_:*FoodItemHealthU*−*FoodItemHealthN* < 0; *mean* = −0.28[−0.78, 0.24], *sd* = 0.31, *p*.*p*. = 0.82, **BF**_**+0**_** = 4.66**).

In addition, we assessed which fixed effect would enhance the predictive capability of the suggested model using PSIS-loo estimations. To check whether PSIS-loo estimations of the compared models are reliable, the full Preference Model $Pareto\;\hat{k}$ values were calculated (Figure 2E). All values were below the suggested (Vehtari et al., 2016; Magnusson et al., 2020) threshold of $Pareto\;\hat{k}<0.7$, ascertaining that the comparison can be trusted. PSIS-loo criteria showed that the addition of PHS and Healthiness would enhance model performance. Although adding Session enhanced PSIS-loo (Table 1), the improvement was below the standard error. Therefore, Preference Model 2 was preferred over the others. This outcome corroborates the results obtained from the full Preference Model, demonstrating that Session and its interactions with Healthiness or PHS did not affect food preferences.

**Table 1:** Fit indices of the preference models computed by multilevel Bayesian cumulative modeling (ordered by fit).

**Preference model**	** ${\hat{elpd}}_{diff}$ **	** $se\left({\hat{elpd}}_{diff}\right)$ **	** ${\hat{elpd}}_{loo}$ **	** $se\left({\hat{elpd}}_{loo}\right)$ **
Model 1: Session*PHS*Healthiness +*RE*	0.0	0.0	**–**58558.7	119.4
Model 2: PHS*Healthiness+**RE**	**–**2.4	4.1	**–**58561.1	119.3
Model 3: *Healthiness*+*RE*	**–**279.1	26.0	**–**58837.8	117.6
Model 4: 1+*RE*	**–**5307.6	97.0	**–**63866.3	70.8

The endorsed model is indicated in bold. All models are compared to the best model (i.e., Model 1). More complex models are considered to be better if they show more than one standard error enhancement in ${\hat{elpd}}_{diff}$ (Vehtari et al., 2016; Magnusson et al., 2020). RE, Random effects. For a detailed specification of the models, see Equations (1)–(4). ${\hat{elpd}}_{loo}$, Expected log pointwise predictive density for a new dataset using the Pareto smoothed importance sampling (PSIS) leave-one-out cross-validation (loo) criterion (Vehtari et al., 2016; Magnusson et al., 2020). The closer to zero, the better the model is. ${\hat{elpd}}_{diff}$, The difference between ${\hat{elpd}}_{loo}$of two compared models. se, Standard error of the targeted variable.

### 3.2. Online supermarket task

**Figure 4:**
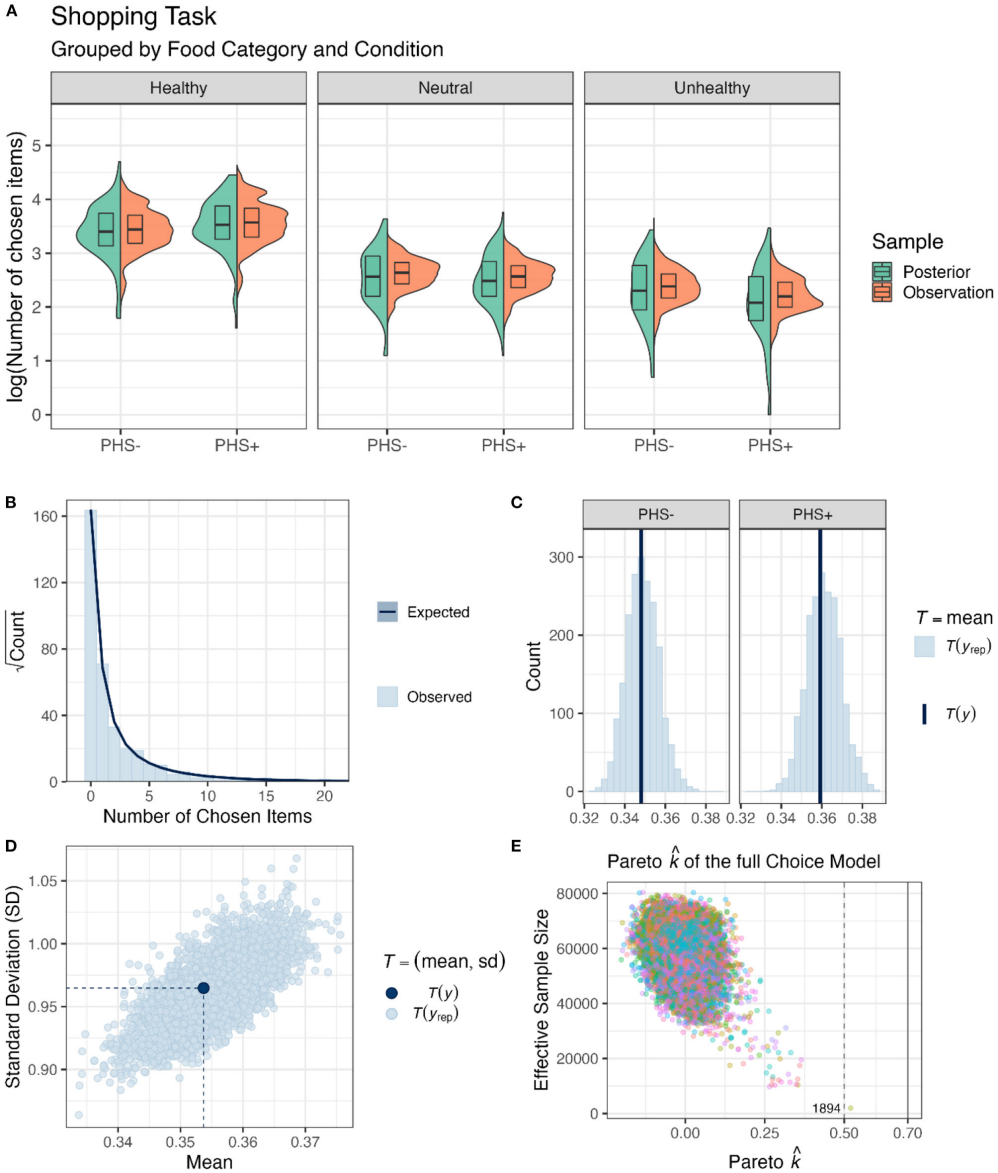
Food choice behavior in the online supermarket task. **(A)** Box and violin plots of the average sum of chosen items per participant, food category, and PHS condition on the log scale (pooled over Session). Note that the data was curated for plotting purposes, as first, the choices were averaged for each health category and participant, and then the log of these values was plotted here. **(B)** A bar plot of the data obtained from the shopping task depicting the distribution of the observations. The dark blue line and shadows represent mean and standard error estimates obtained from posterior distributions. **(C)** Frequency density bar plots showing the estimated (*y*_*rep*_) and observed mean numbers of items chosen (*y*) in the two PHS conditions. **(D)** A scatter plot showing the diffusion of choices. Light blue dots (*y*_*rep*_) are estimated based on posterior distributions, and the dark blue dot (*y*) is the observed value. **(E)** Pareto $\hat{k}$ values for the full Choice Model are depicted against the effective sample sizes for importance sampling. If leaving out an observation changes the posterior too much, then PSIS-loo is not able to give a reliable estimate (Vehtari et al., 2016; Magnusson et al., 2020). However, in the current model and sample, there was no value over 0.7 and only one value over 0.5, which is annotated with the pseudo-ID.

To understand the effects of PHS, Session, and Healthiness on food choices in a realistic shopping simulation, we analyzed the results of the online supermarket task (Figure 4A) by applying the models specified in the Section 2.4. Data analysis. Notably, in the choice models, the number of chosen items per food item (i.e., a discrete-continuous variable) was used as the outcome. The full Choice Model (Equation 1) showed no divergent transition, all *Rhat* = 1.00, and all variables had bulk- and tail-effective sample sizes >2,000 and >5,000. Posterior checks showed that the negative binomial model simulations reasonably capture the features of the observed data, including distributions (Figure 4B), means of conditions (Figure 4C), and dispersion (Figure 4D).

**Figure 5:**
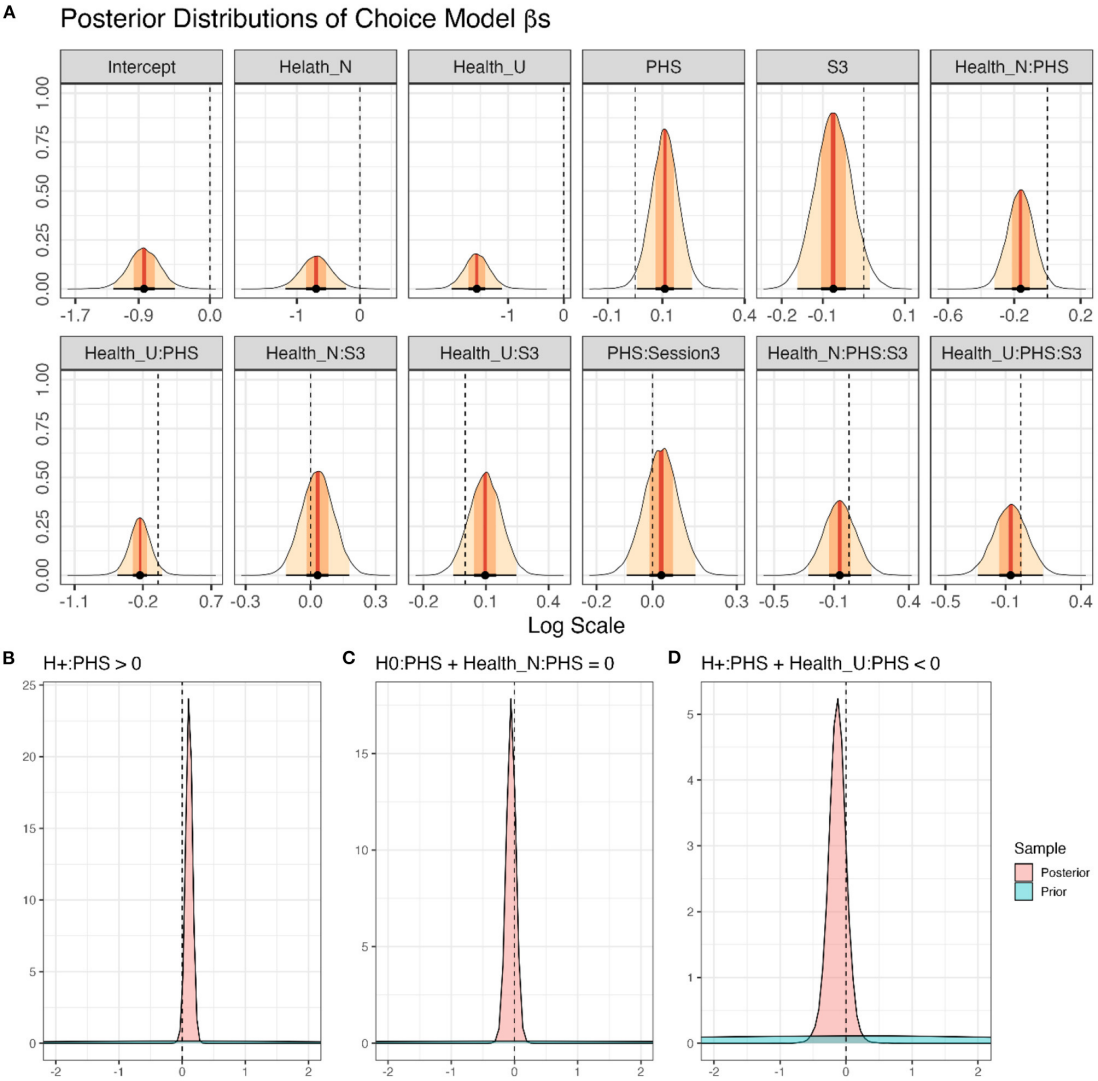
Modeling of choice performance in the online supermarket task. **(A)** Density plots of posterior distributions of all β-coefficients included in the full Choice Model (Equation 1); presented values are in log scale. Red lines and orange and yellow shadows represent the mean point estimates and the 50 and 95% highest probability density (HPD) areas, respectively. Please note the different scalings of the X-axes. **(B–D)** The posterior distributions for specific a priori hypotheses. For more details regarding the hypotheses and results, see the text.

After confirming the validity of the full Choice Model, the posteriors drawn from it were used to test our hypotheses (Figure 5). The results show that PHS increased choices of healthy food items (*H*_+_:*ConditionPHS*>0; *mean* = 0.11[0.02, 0.19], *sd* = 0.05, *p*.*p*. = 0.98, **BF**_**+0**_** = 49.70**). In contrast, PHS did not affect the choices of neutral items (*H*_0_:*ConditionPHS*+ *FoodItemHealthN*:*ConditionPHS* = 0; *mean* = −0.05[−0.19, 0.08], *sd* = 0.07, *p*.*p*. = 0.98, **BF**_**01**_** = 39.57**). Finally, PHS probably decreased choices of unhealthy food items (*H*_+_:*ConditionPHS*+ *FoodItemHealthU*:*ConditionPHS* < 0; *mean* = −0.13[−0.35, 0.09], *sd* = 0.14, *p*.*p*. = 0.85, **BF**_**+0**_** = 5.56**). Similar to preference ratings, even though the results indicated that choices of healthy food items were increased by PHS, the evidence supporting the decrease in choices of unhealthy food items by PHS was not strong. Given that zero is included in the 95% confidence interval of the change in unhealthy food choices due to PHS, one should interpret this result with caution. That is, although this result indicates that choices for unhealthy food items were more likely (*p*.*p*. = 0.85) to decrease rather than increase (*p*.*p*. = 0.15) due to PHS, one should not rule out the possibility of no modulation.

As for preferences, Session did not affect food choice behavior, neither as a main effect (*H*_0_:*SessionS*3 = 0;*mean* = −0.07[−0.16, 0.01], *sd* = 0.04, *p*.*p*. = 0.94, **BF**_**01**_** = 14.42**), nor in interaction with PHS (*H*_0_:*SessionS*3:*ConditionPHS* = 0; *mean* = 0.03[−0.09, 0.15], *sd* = 0.06, *p*.*p*. = 0.97, **BF**_**01**_** = 36.02**), food category (*H*_0_:*SessionS*3:*FoodItemHealthN*+*SessionS*3:*FoodItemHealthU* = 0; *mean* = 0.13[−0.12, 0.37], *sd* = 0.12, *p*.*p*. = 0.94, **BF**_**01**_** = 16.76**), or in interaction with both factors (*H*_0_:*SessionS*3:*ConditionPHS*:*FoodItemHealthN*+*SessionS*3:*ConditionPHS*:*FoodItemHealthU* = 0; *mean* = −0.13[−0.48, 0.22], *sd* = 0.18, *p*.*p*. = 0.94, **BF**_**01**_** = 15.71**).

Finally, healthy food items were chosen more often than both neutral items (*H*_+_:*FoodItemHealthN* < 0; *mean* = −0.70[−1.10, −0.30], *sd* = 0.24, *p*.*p*.>0.99, **BF**_**+0**_** = 299.75**), and unhealthy items (*H*_+_:*FoodItemHealthU* < 0; *mean* = −1.56[−1.94, −1.19], *sd* = 0.23, *p*.*p*.>0.99, **BF**_**+0**_**>9999**). Also, neutral food items were chosen more often than unhealthy ones (*H*_+_:*FoodItemHealthU*−*FoodItemHealthN* < 0; *mean* = −0.87[−1.20, −0.53], *sd* = 0.20, *p*.*p*.>0.99, **BF**_**+0**_**> 9999**).

Additionally, we assessed which fixed effect would enhance the predictive capability of the suggested model using PSIS-loo estimations. To check whether PSIS-loo estimations of the compared models are reliable, the full Choice Model $Pareto\;\hat{k}$ values were calculated (Figure 4E). All values were below the suggested (Vehtari et al., 2016; Magnusson et al., 2020) threshold of $Pareto\;\hat{k}<0.7$, indicating that the comparison can be trusted. PSIS-loo criteria showed that adding PHS and Healthiness enhanced the model performance. However, adding Session deteriorated PSIS-loo (Table 2). Therefore, Choice Model 2 was considered the preferred model. This outcome corroborates the results obtained from the full Choice Model, showing that Session and its interactions did not affect food choices.

**Table 2:** Fit indices of the choice models computed by multilevel Bayesian generalized linear modeling (ordered by fit).

**Choice model**	** ${\hat{elpd}}_{diff}$ **	** $se\left({\hat{elpd}}_{diff}\right)$ **	** ${\hat{elpd}}_{loo}$ **	** $se\left({\hat{elpd}}_{loo}\right)$ **
Model 2: PHS*Healthiness +**RE**	0.0	0.0	−20476.4	174.2
Model 1: Session*PHS*Healthiness+*RE*	−3.8	2.5	−20480.2	174.3
Model 3: *Healthiness*+*RE*	−79.9	5.8	−20556.2	174.3
Model 4: 1+*RE*	−4754.3	100.2	−25230.6	208.0

The endorsed model is indicated in bold. All models are compared to the best model (i.e., Model 2). More complex models are considered to be better if they show more than one standard error enhancement in ${\hat{elpd}}_{diff}$ (Vehtari et al., 2016; Magnusson et al., 2020). RE, Random effects. For a detailed specification of the models, see Equations (1)–(4). ${\hat{elpd}}_{loo}$, Expected log pointwise predictive density for a new dataset using the Pareto smoothed importance sampling (PSIS) leave-one-out cross-validation (loo) criterion (Vehtari et al., 2016; Magnusson et al., 2020). The closer to zero, the better the model is. ${\hat{elpd}}_{diff}$, The difference between ${\hat{elpd}}_{loo}$of two compared models. se, Standard error of the targeted variable.

### 3.3. Choice-preference function

**Figure 6:**
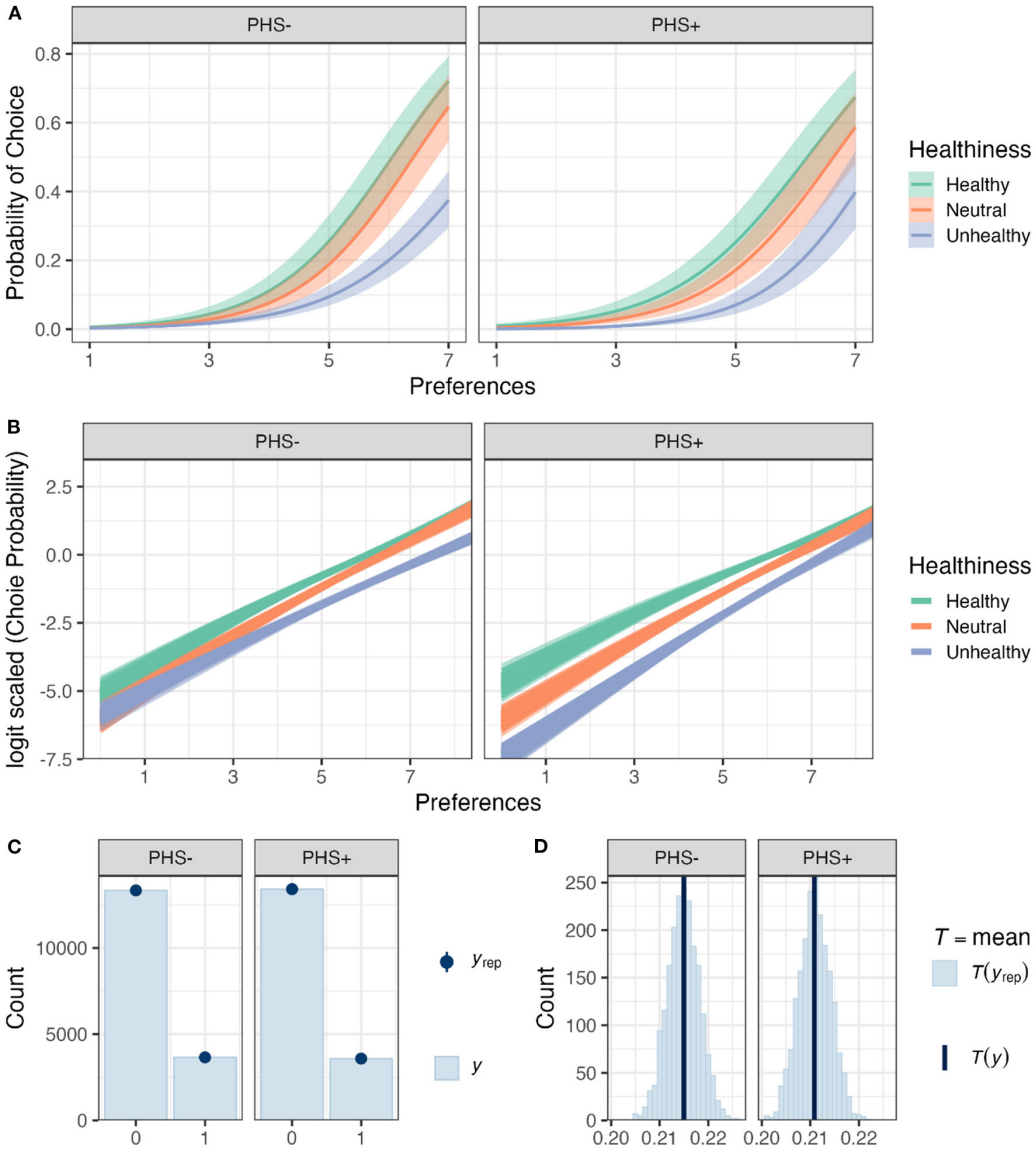
The relationship of food preferences and choice behavior in the supermarket task. **(A)** Choice probabilities as a function of preferences based on model predictions for different food categories and PHS conditions. Solid lines and shadows represent means and standard errors based on posterior distributions, respectively. **(B)** Choice probabilities in logit scale as a function of preferences based on model predictions for different food categories and PHS conditions. In total, 200 lines are shown for each condition, where each line presents a posterior prediction. **(C)** A bar plot (*y*) of the data obtained from the choice behavior (i.e., the model's outcome) depicting the distribution of the observations. The blue dots and lines (*y*_*rep*_) represent mean and standard error estimates obtained from posterior distributions of the full Choice-Preference Function. **(D)** Frequency density bar plots showing the estimated (*y*_*rep*_) and observed mean choice behavior (*y*) in the two PHS conditions.

After analyzing the online supermarket and subjective rating tasks separately, we addressed whether the relationship between choice behavior and preferences was modulated by PHS, Session, and Healthiness of the food items. For this purpose, the choice-preference function was calculated (logistic regression hierarchical Bayesian models), where binary choices were modeled by using preferences as a regressor (Figure 6A). The full Choice-Preference Function (Equation 6) showed no divergent transitions, all *Rhat* = 1.00, and all variables had bulk- and tail-effective sample sizes >3,000 and >6,000. Posterior checks showed that the logistic model simulations reasonably captured the features of the observed data, including distributions (Figure 6C) and means of different conditions (Figure 6D).

**Figure 7:**
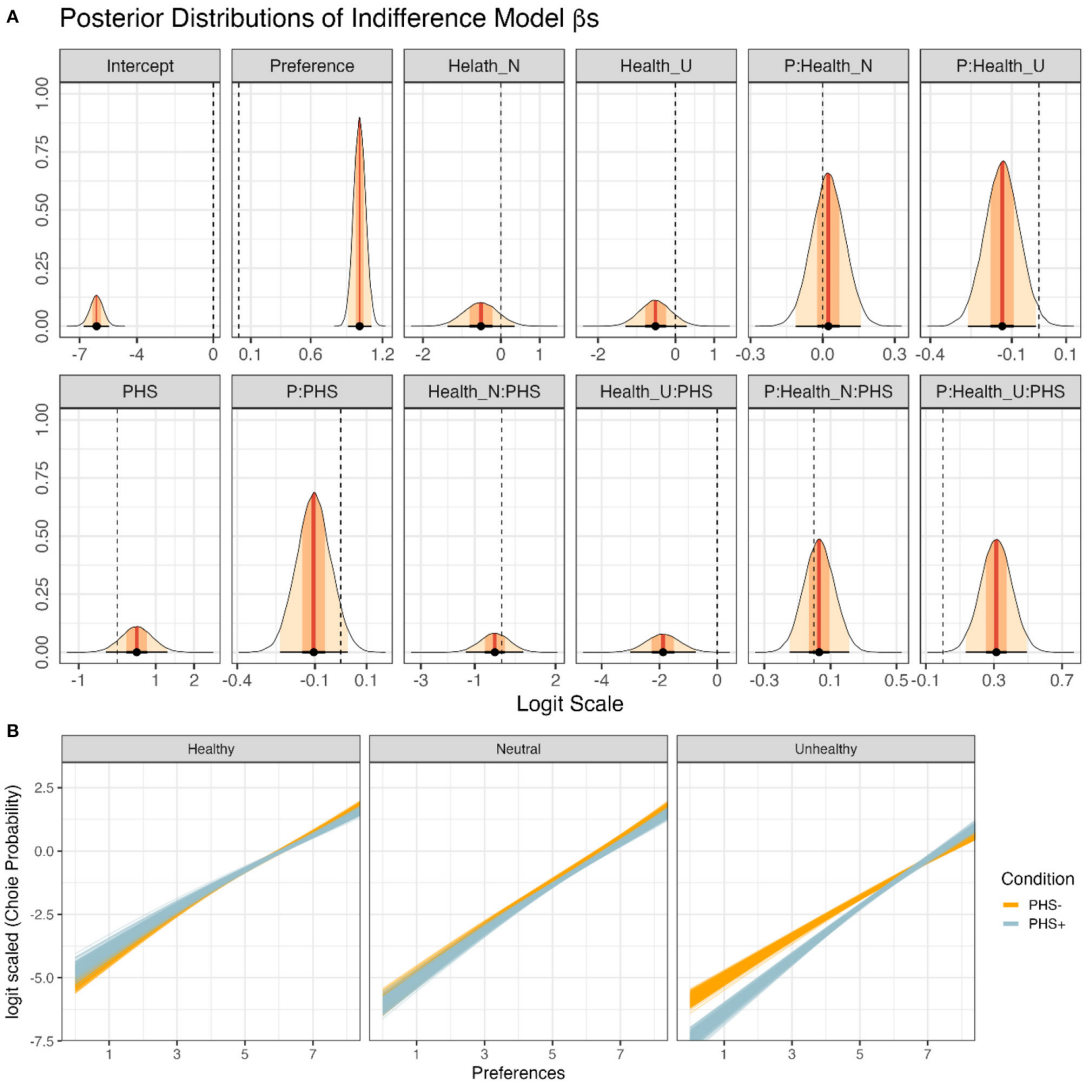
Modeling of the relationship between choices, as measured by the online supermarket task, and preferences, as measured by the subjective rating task. **(A)** Posterior distributions of all β-coefficients included in the full Preference Model, except those containing Session or its interactions (Equation 6). Red lines and orange and yellow shadows represent the mean point estimates and the 50 and 95% highest probability density (HPD) areas, respectively. Please note the different scalings of the X-axes. **(B)** The posterior predictions for specific hypotheses. For more details regarding the hypotheses and results, see the text. Each line presents a posterior prediction; in total, 200 predictions are shown.

After confirming the validity of the full Choice-Preference Function, the posteriors drawn from it were used to test our hypotheses (Figure 7A). Two sets of results are presented; the first set is related to the interaction of the experimental factors with preferences, which are associated with the modulation of slopes of the *Choice*~*Preference* relationship (Figure 6B). These results indicate the importance of preferences in choice behavior. The second set is related to the effects of the experimental factors on choices regardless of preferences, which is represented by the intercepts of the *Choice*~*Preference* relationship (Figure 6B). These results reveal general biases toward choosing items of a certain category in different conditions, regardless of preferences.

First, we focused on the effects of preferences on choice behavior in the different food categories (Figure 7B). For healthy items, increased preferences positively affected choices (*H*_+_:*Preferences*>0; *mean* = 1.01[0.93, 1.09], *sd* = 0.05, *p*.*p*.>0.99, **BF**_**+0**_**>9999**). The relationship between preferences and choices was indistinguishable between the neutral and healthy food categories (*H*_0_:*Preferences*:*FoodItemHealthN* = 0; *mean* = 0.02[−0.11, 0.16], *sd* = 0.07, *p*.*p*. = 0.97, **BF**_**01**_** = 34.35**. Interestingly, the importance of preferences in choice behavior was less pronounced for unhealthy compared to healthy food items (*H*_+_:*Preferences*:*FoodItemHealthU* < 0; *mean* = −0.14[−0.24, −0.03], *sd* = 0.06, *p*.*p*. = 0.98, **BF**_**+0**_** = 61.31**).

Further, participants were more likely to show a negative bias (i.e., diminished choice behavior regardless of preferences) for both neutral (*H*_+_:*FoodItemHealthN* < 0; *mean* = −0.51[−1.23, 0.21], *sd* = 0.44, *p*.*p*. = 0.88, **BF**_**+0**_** = 7.18**) and unhealthy items (*H*_+_:*FoodItemHealthU* < 0; *mean* = −0.51[−1.18, 0.16], *sd* = 0.41, *p*.*p*. = 0.88, **BF**_**+0**_** = 8.32**) as compared to healthy food items. However, there was no difference in choice bias for unhealthy compared to neutral items (*H*_0_:*FoodItemHealthU*−*FoodItemHealthN* = 0; *mean* = 0.0[−0.81, 0.80], *sd* = 0.42, *p*.*p*. = 0.89, **BF**_**01**_** = 8.23**).

Second, we addressed whether PHS had altered the relationship between choice behavior and preferences (i.e., slopes) for the different food categories. Notably, PHS did not affect the relationship between choices and preferences for both healthy items (*H*_0_:*Preferences*:*ConditionPHS* = 0; *mean* = −0.10[−0.23, 0.03], *sd* = 0.07, *p*.*p*. = 0.92, **BF**_**01**_** = 11.24**) as well as neutral items (*H*_0_:*Preferences*:*ConditionPHS* + *Preferences*:*FoodItemHealthN*:*ConditionPHS* = 0; *mean* = −0.07[−0.21, 0.06], *sd* = 0.07, *p*.*p*. = 0.97, **BF**_**01**_** = 28.43**). In contrast, unhealthy items were more likely to be affected by preferences when PHS was activated compared to deactivated (*H*_+_:*Preferences*:*ConditionPHS* + *Preferences*:*FoodItemHealthU*:*ConditionPHS*>0; *mean* = 0.21[0.10, 0.32], *sd* = 0.07, *p*.*p*.>0.99, **BF**_**+0**_** = 1110.11**).

Also, when considering choice biases (i.e., intercepts), PHS+ compared to PHS- did not affect choice bias for healthy (*H*_0_:*ConditionPHS* = 0; *mean* = 0.51[−0.29, 1.31], *sd* = 0.41, *p*.*p*. = 0.92, **BF**_**01**_** = 11.24**) or neutral items (*H*_0_:*ConditionPHS* + *FoodItemHealthN*:*ConditionPHS* = 0; *mean* = −0.07[−0.21, 0.06], *sd* = 0.07, *p*.*p*. = 0.97, **BF**_**01**_** = 28.43**). In contrast, PHS+ induced a negative bias for unhealthy items compared to PHS- (*H*_+_:*ConditionPHS* + *FoodItemHealthU*:*ConditionPHS* < 0; *mean* = −1.37[−2.10, −0.65], *sd* = 0.44, *p*.*p*.>0.99, **BF**_**+0**_** = 753.72**).

Together, the results indicate that PHS affected the relationship between preferences and choices only for unhealthy food items (Figure 7B). These effects, however, are two-fold. PHS caused participants to reject unhealthy items more frequently regardless of preferences (i.e., a negative choice bias). Simultaneously, PHS made preferences more critical in participants' unhealthy choices (Figure 7B).

Finally, we checked which fixed effect would enhance the predictive capability of the suggested model using PSIS-loo estimations. To check whether PSIS-loo estimations of the compared models are reliable, the full Choice-Preference Function $Pareto\;\hat{k}$ values were calculated. All values were below the suggested (Vehtari et al., 2016; Magnusson et al., 2020) threshold (i.e., $Pareto\;\hat{k}<0.7$), ascertaining that the comparison can be trusted. PSIS-loo criteria showed that the addition of PHS and Healthiness enhanced model performance. However, adding Session deteriorated PSIS-loo (Table 3). Therefore, the Choice-Preference Function 2 was considered the preferred model, suggesting that Session and its interactions did not affect choice-preference relationships.

**Table 3:** Fit indices of the choice-preference functions computed by multilevel Bayesian logistic regression linear modeling (ordered by fit).

**Choice-preference function**	** ${\hat{elpd}}_{diff}$ **	** $se\left({\hat{elpd}}_{diff}\right)$ **	** ${\hat{elpd}}_{loo}$ **	** $se\left({\hat{elpd}}_{loo}\right)$ **
Model 2: Preferences*PHS*Healthiness+RE	0.0	0.0	−10600.2	101.2
Model 1: Preferences*Session*PHS*Healthiness+*RE*	−2.1	4.6	−10602.3	101.3
Model 3: Preferences*Healthiness+*RE*	−44.5	10.3	−10644.8	102.2
Model 4: *Preferences*+*RE*	−1574.4	55.3	−12174.7	100.3

The endorsed model is indicated in bold. All models are compared to the best model (i.e., Model 2). More complex models are considered to be better if they show more than one standard error enhancement in ${\hat{elpd}}_{diff}$ (Vehtari et al., 2016; Magnusson et al., 2020). RE, Random effects. For a detailed specification of the models, see Equations (1)–(4). ${\hat{elpd}}_{loo}$: Expected log pointwise predictive density for a new dataset using the Pareto smoothed importance sampling (PSIS) leave-one-out cross-validation (loo) criterion (Vehtari et al., 2016; Magnusson et al., 2020). The closer to zero, the better the model is. ${\hat{elpd}}_{diff}$, The difference between ${\hat{elpd}}_{loo}$, of two compared models. se, Standard error of the targeted variable.

### 3.4. Hypnotizability

Two approaches were used to address whether hypnotizability scores predict changes in subjective ratings and choice behavior. First, the full models (Equation 1) were compared with models that did not contain random slopes but were otherwise identical to the full model. Second, a robust Bayesian correlation test was conducted to understand whether the changes in healthy, neutral, or unhealthy categories due to PHS were correlated with hypnotizability scores.

**Figure 8:**
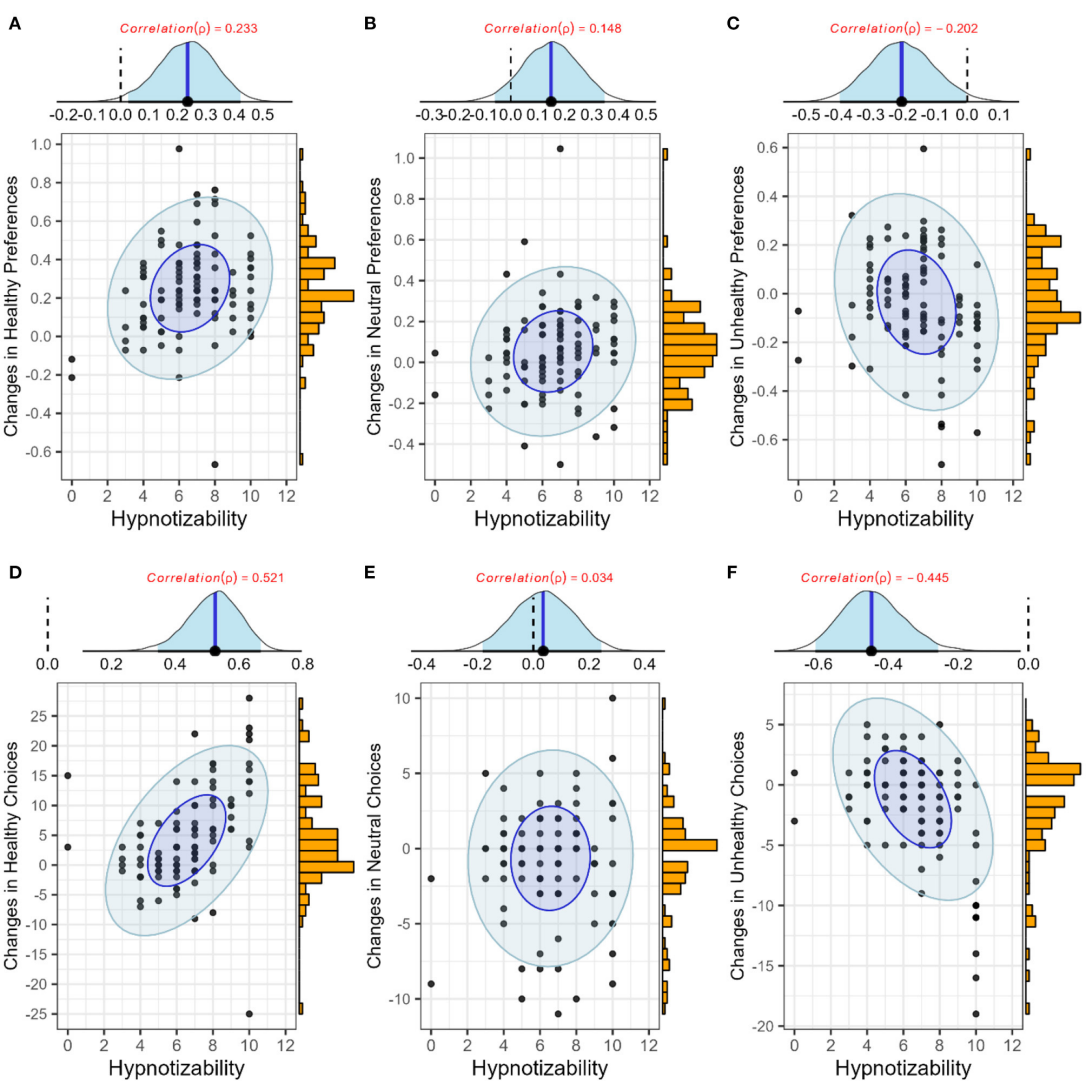
Effects of hypnotizability on Food Preferences (top) and Food Choice (bottom). Each panel shows three elements. (Top) The posterior distribution of ρ (blue lines and shadows show means and 95% highest density intervals); (right) a histogram of changes in the targeted variable drawn from observations; and (mid) point and eclipse plots, representing the observations and robust posterior predictions, respectively. Dark blue and light blue ellipses show the 50 and 95% highest density areas. **(A–C)** Changes in preferences for healthy, neutral, and unhealthy items; **(D–F)** Changes in choices of healthy, neutral, and unhealthy items.

Regarding subjective preferences, the model without random slope was significantly worse than the full model (${\hat{elpd}}_{diff}=\;-237.4$, $se({\hat{elpd}}_{diff})$ = 24.1, **BF**_**10**_**>999**), showing that hypnotizability scores are crucial for predicting participants' preferences. Further, the robust correlation test corroborated this result (Figures 8A–C), revealing that changes in preferences for healthy (ρ_*mean*_ = 0.232 [ 0.030, 0.423], *sd* = 0.100, **p.p**_**ρ>0**_** = 0.98**) and unhealthy items (ρ_*mean*_ = −0.202 [ −0.400, −0.010], *sd* = 0.100, **p.p**_**ρ < 0**_** = 0.97**) were correlated with hypnotizability scores positively and negatively, respectively. However, the changes in preferences for the neutral food category were not significantly correlated with hypnotizability (ρ_*mean*_ = +0.148 [ −0.058, 0.347], *sd* = 0.104, *p*.*p*_ρ>0_ = 0.88).

Also, regarding choice behavior in the shopping task, the model without random slope was significantly worse than the full model (${\hat{elpd}}_{diff}=\;-66.4$, $se({\hat{elpd}}_{diff})$ = 12.8, **BF**_**10**_**>999**), showing that hypnotizability is crucial for predicting participants' choices. Additionally, the robust correlation test corroborated this result (Figures 8D–F), revealing that changes in choosing healthy (ρ_*mean*_ = 0.520 [0.358, 0.677], *sd* = 0.081, **p.p**_**ρ>0**_**>0.99**) and unhealthy items (ρ_*mean*_ = −0.444 [−0.620, −0.272], *sd* = 0.090, **p. p**_**ρ < 0**_** = 0.99**) were correlated positively and negatively with hypnotizability, respectively. However, the changes in neutral food category choices were not significantly correlated with hypnotizability (ρ_*mean*_ = 0.033 [ −0.182, 0.243], *sd* = 0.109, *p*.*p*_ρ>0_ = 0.62).

Finally, for the choice-preference function, we compared the full models (Equation 6) with models without random slopes but otherwise identical to the full model. The model without random slope was significantly worse than the full model (${\hat{elpd}}_{diff}=\;-23.9$, $se({\hat{elpd}}_{diff})$ = 7.1, **BF**_**10**_**>999**), revealing that hypnotizability scores are crucial for predicting the choice-preference function. Notably, as slopes and choice biases derived from the choice-preference function were the prediction of the Bayesian logistic model, using them for modeling the robust Bayesian correlation might have been misleading; therefore, we refrained from using this approach.

## 4. Discussion

To address the effects of PHSs on food preferences and choices, we conducted an online-only, repeated-measures study with three sessions. In the first session, participants' hypnotizability was measured using HGSHS. Notably, all participants were included in the sample regardless of their hypnotizability. At the beginning of Session 2, participants received hypnosis, including a PHS aiming to increase preferences for healthy food items. Following the hypnosis, they took part in our task set twice, once when the PHS was activated and once when it was deactivated. The task set consisted of a subjective rating task, measuring participants' explicit preferences for a large number of diverse food items, and a realistic shopping simulation, measuring participants' choice behavior for the same items. Session 3 mimicked Session 2 but did not repeat hypnosis and PHS instructions but merely activated and deactivated the PHS introduced in Session 2.

The results of the rating task revealed that the PHS increased preferences for healthy food items. These results are in line with previous reports, showing that PHSs can successfully alter food preferences (Ludwig et al., 2014; Zahedi et al., 2020a). Notably, the results of our online supermarket task revealed that the increase in explicit preferences for healthy items was accompanied by increased choices in a realistic shopping scenario. This finding is of great importance when considering the prevalence of obesity in industrialized societies (Swinburn et al., 1999; Jaacks et al., 2019) and the inability of traditional approaches, such as diet control and cognitive training, to change patterns of food consumption (for review, see Stephens et al., 2014; Yang et al., 2019).

The choice-preference function analysis showed that although the PHS affected food preferences and choices of healthy items, the relationship between preferences and choices was not altered. In other words, one may reasonably suggest that changes in choice behavior in favor of healthy items were driven by increased preferences for these types of food. As Ainslie (2020) discussed, this form of choice modulation, called resolve, can be distinguished from changes in choice behavior by means of suppressing unaltered preferences. Although resolve is a subcomponent of inhibition, a well-investigated executive function (Miyake et al., 2000; Miyake and Friedman, 2012; Diamond, 2013; Yuan and Raz, 2014; Limbers and Young, 2015), there is an ongoing debate about the efforts involved in its implementation (Ainslie, 2020). Considering an example clarifies this point. If someone has a strong preference for food item A but should not or does not want to consume it for any reason, there are two options to change their behavior: suppression and resolve. Suppression refers to refraining from consuming food item A, even though it is preferred, which requires constant effort. In contrast, resolve refers to following an alternative, possibly preconceived plan for selecting an alternative food item B. One reasonable strategy is to associate food Item B with positive rewards and item B with negative concepts, or in other words, attenuate the comparative subjective value of item A. As argued by Ainslie (2020), suppression is effortful, while resolve may not be. Certainly, it will be highly valuable to address the required efforts in inhibition involving mere suppression vs. inhibition relying on resolve.

Notably, a number of studies have shown that (post-)hypnotic suggestions are implemented through top-down modulations (Terhune et al., 2017), require attention allocation (Tobis and Kihlstrom, 2010), and require cognitive effort (Parris et al., 2021). Further, the relationship between food-related PHSs and executive functions (requiring cognitive effort) has also been demonstrated (Ludwig et al., 2014; Zahedi et al., 2020a). Combining our current results with previous findings regarding the effortfulness of PHS effects (Tobis and Kihlstrom, 2010; Zahedi et al., 2017, 2019, 2020a), we tentatively suggest that resolve, like its sibling suppression, might be effortful at the time of implementation (Zahedi et al., 2020a). However, unlike suppression, resolve leads to long-term changes in choice behavior through modulation of preferences that may be implemented effortlessly (Ainslie, 2020). One should note that these interpretations are speculative and require further investigations.

The PHS not only increased the preference for healthy items but also decreased preferences and choices of unhealthy food items. For unhealthy items, the choice-preference function analysis revealed that—in contrast to healthy and neutral items—the PHS altered the relationship between food choices and preferences. The effects were two-fold. First, the PHS induced a negative choice bias for unhealthy items, meaning when disregarding the effects of preferences, participants were less likely to choose unhealthy items when the PHS was activated. However, food preferences became more critical in unhealthy choices when the PHS was activated compared to the deactivated condition. When interpreting these results, one should consider two points. (I) As the current study's PHS (Appendix A) focuses on healthy items, one might suggest that changes in other food categories, such as unhealthy items, indicate demand characteristics rather than genuine modulation of choice behavior. Bayesian statistics (van Doorn et al., 2021) showed that for neutral food items, neither separately analyzed preferences and choices nor the relationship between them were affected by the PHS. If participants were responding to demand characteristics (e.g., being positively biased toward healthy items), one should also expect a decrease in the preferences and choices of neutral items. Therefore, the changes in choices of unhealthy food items are hard to explain in terms of demand characteristics. (II) Food preferences are not the only factor affecting food choices. Other factors, such as impulsivity (Guerrieri et al., 2008; Wiers et al., 2011; Jones et al., 2018) and transitory states like hunger or stress (Nederkoorn et al., 2009; Froehlich et al., 2021a,b), can strongly affect food choices. Therefore, a plausible explanation for the changes observed regarding unhealthy food items is that under the effects of the PHS, participants were more thoughtful regarding unhealthy choices, with the consequence of suppressing these choice options more frequently.

The deliberate decision-making strategy employed by participants regarding unhealthy food items can be contrasted with being impulsive (Pereira et al., 2005; Navarro-Allende et al., 2008; Kakoschke et al., 2017; Jones et al., 2018). Additionally, however, it should be contrasted to the changes in healthy food choices, which may be accounted for by implementing “resolve.” Specifically, since previous findings showed that participants are better at inhibiting temptations by unhealthy food under the effects of PHSs (Zahedi et al., 2020a), this interpretation seems even more justified. Given that in the obesogenic environments governing most industrialized countries (Swinburn et al., 1999; Jaacks et al., 2019), the ever-increasing influence of impulsive behaviors might play a significant role in unhealthy food choices (Pereira et al., 2005; Navarro-Allende et al., 2008; Kakoschke et al., 2017; Jones et al., 2018), PHSs might be an important tool for fighting the obesity epidemic. Another translational value of our results is related to the unsustainability of unhealthy food choices from the environmental perspective (Clark et al., 2019, 2022; Willett et al., 2019). Hence, the observed increased preferences for healthy food items and decreased choices from the unhealthy category are crucial not only for human health but also for planetary sustainability. However, these results and interpretations need to be replicated by other groups and further investigated before one can draw any conclusion with certainty.

Interestingly, our Bayesian results confirmed that the observed effects of the PHS were not diminishing over a period of more than 1 week. Even though some anecdotal reports suggest the longevity of PHS effects (for review, see Zahedi et al., 2017; Bohmer and Schmidt, 2022), few studies have investigated the question. For instance, Bohmer and Schmidt (Bohmer and Schmidt, 2022) have shown that a safety-promoting PHS was effective over several weeks (*Median* = 49 *days, Range* = 7169 *days*) after hypnosis induction. In line with previous reports, our results not only show the longevity of PHS effects but also revealed that these effects are not qualitatively or quantitatively altered. This point has important implications for theories of hypnosis. For instance, it is suggested that the effects of PHSs might be implemented through context-dependent mental practice (Zahedi et al., 2020b). Our results corroborate this hypothesis, as the effects neither diminished nor increased in the absence of renewed hypnosis and PHS. Further, the effects were still confined to a specific context (activation signals), even a week after receiving the PHS.

Another facet of our results critical for hypnosis theories is that changes in preferences and choice behavior for both healthy and unhealthy food categories correlated with participants' hypnotizability scores. Given that hypnotizability itself is a multifactorial construct (Woody et al., 2005; Zahedi and Sommer, 2022), many active researchers in the hypnosis field suggested that participant selection should not be based on hypnotizability (Jensen et al., 2015; Acunzo and Terhune, 2021; Reshetnikov and Terhune, 2022; Zahedi and Sommer, 2022). Our results of a robust relationship between PHS effects and hypnotizability echo these suggestions and indicate the value of using hypnotizability as a regressor for modeling results rather than as a cut-off criterion in participant selection. According to the present findings, even a simple suggestion might have an intricate range of effects implemented *via* different psychological mechanisms. Therefore, the current study strongly suggests that theories of hypnosis, which try to simplify hypnotic phenomena to a single psychological mechanism, are of limited value (Zahedi and Sommer, 2021, 2022; Lynn et al., 2022).

An interesting point regarding our results is the higher preference for healthy food items even when PHS was not activated. This finding is in accordance with other studies, some of which used considerably bigger samples (Blechert et al., 2014; Zahedi et al., 2022). The reason for this initial difference might be related to a multitude of factors (Scaglioni et al., 2018), the discussion of which is outside the scope of the current study. However, regardless of these categorical differences, we found that preferences for different food categories were significantly altered by PHS. When discussing changes in food preferences, we are referring to these significant statistical shifts away from the baseline, which are orthogonal to the comparative structure of preferences for different food categories (e.g., healthy vs. unhealthy food preferences).

Several critical points and limitations should be considered regarding the current study. First, our sample included many female students, which may limit the generalizability of the obtained results. However, qualitatively similar results have been obtained in other studies (Ludwig et al., 2014; Zahedi et al., 2020a), speaking in favor of their stability. Further, we did not introduce budgetary restrictions in the online supermarket task because it might have interacted with or even overshadowed the effects of participants' preferences on their food choices (e.g., Darmon et al., 2002; van Dooren, 2018; Fulgoni III and Drewnowski, 2019). Additionally, there are many other factors that can affect food choices and preferences, including, but not restricted to, genetic and prenatal factors (Maier-Noth, 2019), exposure (Bornstein, 1989), and a multitude of affective (Zahedi et al., 2020a), social, and cultural influences (Enriquez and Archila-Godinez, 2021), which were not included in the current study. The present study's focus was the rather specific question of the efficacy of PHS for altering food preferences and choices and addressing their underlying cognitive mechanisms. Future studies, however, should consider these other factors when investigating food choice behavior using appropriate participant samples. Finally, the present study used a PHS that exclusively targeted healthy food items (Appendix A); therefore, other food categories could have been affected only indirectly. Consequently, future studies should investigate PHSs that (also) target unhealthy food preferences.

In conclusion, the current study used an online-only procedure in a repeated-measures design to address the effects of PHSs on food decisions and their underlying mechanisms. Our results indicate that PHSs can successfully increase preferences and choices of healthy food items in a realistic shopping simulation without altering the relationship between preferences and choices for these items. Hence, the alterations in decision-making were most probably implemented through resolve; in other words, the modulation of preferences resulted in the alteration of choice behavior (Ainslie, 2020). On the other hand, although not directly addressed, preferences for and choices of unhealthy food items were decreased due to the PHS. However, for unhealthy food items, the PHS also modulated the relationship between preferences and choices. Simultaneously, participants became more negatively biased against unhealthy items under the influence of the PHS, but preferences also played a more dominant role in their choices. This result was interpreted as indicating less impulsive unhealthy choices under the influence of the PHS, which were implemented through effortful choice suppression. Further, our results showed qualitative and quantitative persistence of the PHS effects, at least over a period of 1 week, which is in line with other findings (Bohmer and Schmidt, 2022). Finally, our results revealed correlations between the PHS effects and hypnotizability, a multifactorial construct (Zahedi and Sommer, 2022). The present results are not only significant in providing a promising tool for counteracting the overweight and obesity epidemic in modern societies but may also contribute toward greater sustainability of food systems. Furthermore, the results valuably contribute to a better theoretical understanding of hypnosis, hypnotizability, and food decisions in general.

## Data availability statement

The datasets presented in this study can be found in online repositories. The names of the repository/repositories and accession number(s) can be found below: https://doi.org/10.17605/OSF.IO/E8H3Q.

## Ethics statement

The studies involving human participants were reviewed and approved by Ethics Committee of the Department of Psychology of the Humboldt-Universität zu Berlin (approval number 2021-36). The patients/participants provided their written informed consent to participate in this study.

## Author contributions

AB: methods development and manuscript writing. AZ: conceptualization, design, manuscript writing, methodology, and data analysis. JL and RÖ: data acquisition and data pre-analysis. WS: conceptualization, design, and manuscript writing. All authors contributed to the article and approved the submitted version.

## Appendix

### Appendix A: The hypnosis procedure and posthypnotic suggestion

The hypnosis procedure and the posthypnotic suggestion used in this study are similar to (Zahedi et al., 2020a). The hypnosis narration was recorded in German and presented from tape to provide identical wordings for all participants; however, if participants needed further elaboration, some additional suggestions related to relaxation were presented by the experimenter A.Z., a certified hypnotizer who was present in all sessions. These suggestions were either progressive muscle relaxation (PMR), breathing techniques, or other suggestions similar in nature (Hammond, 1990, 1998). Before hypnosis, participants chose either a forest or beach scenario for the following hypnosis narration. The induction and deepening stages of hypnosis (Terhune et al., 2017; Zahedi and Sommer, 2021) were succeeded by a suggestion about feeling a lightness in the body and by the following PHS (translated from German): “While you are responding to the tasks, you will hear the sound of a bell. When you have heard it, you will feel a lightness in your body (bell). The lightness is like what you have sensed before and retained in your fist, but now another feeling also accompanies this lightness, a craving, voracious desire for vegetables, fruits, and all sorts of healthy food. You will have a craving even for pictures of vegetables. Even their picture is so desirable and appealing that it increases your appetite and makes you want to eat them. While you are performing the tasks, whenever you see their picture, your appetite and desire for vegetables and fruits, which are healthy and full of vitamins, will become voracious. This exclusive desire for vegetables and fruits will get stronger and stronger during the session. When you hear the sound of the bell for the second time, everything will go back to normal, like before the first sound of the bell; even your hunger will disappear as if it had never existed; everything will go back to normal.”

The PHS was given twice in a row to consolidate the association with the bell ring. Then, hypnosis was terminated with the countback technique (for details, see Hammond, 1990, 1998).
